# Emotion and magnitude perception: number and length bisection

**DOI:** 10.3389/fnbot.2013.00024

**Published:** 2013-12-16

**Authors:** Sylvie Droit-Volet

**Affiliations:** Laboratoire de Psychologie Sociale et Cognitive, CNRS, UMR 6024, Département de Psychologie, Université Blaise PascalClermont-Ferrand, France

**Keywords:** emotion, bisection, time, number, length

## Abstract

Studies of the effect of emotional stimuli on time perception have shown that a threatening stimulus produces a temporal lengthening effect compared to a non-threatening stimulus. In order to better understand the mechanisms underlying this emotion-related time distortion, the present study examined distortions in the judgment of other quantities – number and length – under the same emotional conditions as those previously used for time. However, the nature of the presentation of quantities was manipulated by using a sequential and a non-sequential presentation. The participants were thus given a number or a length bisection task in a sequential or a non-sequential modality of stimulus presentation. In each condition, the participants completed trials in which the probe stimulus was followed by either an aversive stimulus or a non-aversive stimulus. The results showed that the quantities were judged longer, with the set of dots judged bigger and the line judged longer, on the trials which contained aversive stimulus, but only when these quantities were presented sequentially. In comparison with the time distortions obtained in time bisection, these distortions in the bisection judgment of sequentially presented quantities suggests that emotion affected the dynamic process of accumulation of information in working memory.

## INTRODUCTION

In recent decades, ample evidence has been found that emotions (negative and highly arousing emotions in particular) distort judgments of time in human beings. Researchers have tested a wide variety of threatening stimuli in different temporal tasks: facial expressions of anger or fear (e.g., Thayer and Schiff, 1975; Droit-Volet et al., 2004; Tipples, 2008; Bar-Haim et al., 2010), pictures and sounds from the international affective picture and sound systems (e.g., Angrilli et al., 1997; Grommet et al., 2010; Mella et al., 2010; Gil and Droit-Volet, 2012) or aversive stimuli such as electric shocks or acoustic signals that produce a mild pain in the ears (Falk and Bindra, 1954; Hare, 1963; Droit-Volet et al., 2010). Using these different stimuli, most of these researchers have observed a temporal lengthening effect, thus suggesting that durations are judged longer in response to threatening than to non-threatening stimuli. The aim of our study was to examine whether this emotion-related distortion effect might not be specific to time and can instead also be observed in the evaluation of other quantities, such as numerosity or length.

According to the theory of magnitude (ATOM; Walsh, 2003; Bueti and Walsh, 2009), the processing of time, number and length is underpinned by shared mechanisms. This idea originates in behavioral data showing that the discrimination of all quantities obeys Weber’s law (for a recent publication on this topic, see Dehaene and Brannon, 2011). Indeed, estimates of all quantities are accurate on average, and their variability (*SD*) increases in proportion to the amplitude of the magnitudes. However, one specific characteristic of time compared to other magnitudes is that it is a continuous variable whose processing involves a dynamic system that is able to capture the continuous flow of incoming information and retain this in memory. In the internal clock models of the scalar expectancy theory (Gibbon, 1977; Gibbon et al., 1984), this system is described as an accumulator that counts the temporal units (pulses) emitted by a pacemaker during the processing of the overall duration. The total number of temporal units is then transferred and held in a memory system until the final judgment is made. However, some researchers have cast doubt on the idea that there is an accumulator distinct from the working memory system (Ivry and Schlerf, 2008; Block et al., 2010) since the role of working memory is precisely to allocate attentional resources to the processing of information and to maintain this information in short-term memory (Baddeley and Hitch, 1974). The specificity of the processing of temporal information, which is a dynamic dimension, thus lies essentially in a sustained process of accumulation of incoming information in working memory.

The mechanisms underlying the time distortions observed under the effect of threatening stimuli are currently a subject of debate (for a recent review, see Droit-Volet et al., 2013). The most widely proposed explanation is that the increase in arousal level in a threatening context increases the speed of a time-specific system (i.e., the pacemaker of the clock). When the internal clock speeds up, more units are accumulated and time is judged to be longer. However, within the theoretical framework of a system common to the three magnitudes of time, number and length, we can suppose that emotion would also affect the judgment of quantities other than time. This is consistent with the results found by Jones et al. (2011) who showed that a 5-s click train that produced a lengthening effect on time judgments also improved performance in other tasks such as item recall. However, Droit-Volet (2010) found that this type of click train, which produced a lengthening effect in a timing task, produced a lengthening effect for the discrimination of other quantities (i.e., number and length), but only when these quantities were presented sequentially. In the non-sequential presentation, the participants judged the numerosity of a set of dots or the length of a line. In the sequential presentation, they judged the total number of dots in a series of dots (presented successively) or the total length of a series of small lines. The specificity of the processing of sequentially presented quantities is that it requires the units (dots, lines) to be accumulated in memory. The participants must indeed add together the units and retain the result in memory while they capture the next units. This dynamic process of accumulating units in working memory is similar to that required when processing the flow of temporal information. Consequently, we can assume that high-arousing emotions affect the information accumulation mechanism in working memory, which is common to continuous quantities, rather than boosting the rate of a time-specific mechanism. Our hypothesis is thus that a highly arousing emotion should produce a “lengthening” effect in number and length discrimination tasks, but only when the quantities are presented sequentially. In line with this hypothesis, a recent study using emotional facial expressions in temporal bisection and numerosity bisection replicated the lengthening effect for time but not for numbers which were presented non-sequentially (i.e., a set of dots; Young and Cordes, 2012).

The aim of the present study was therefore to examine whether a threatening situation produces a distortion in the discrimination of number and length as has previously been found for the discrimination of time. The threatening stimulus in our study took the form of an aversive stimulus that has already been shown by Droit-Volet et al. (2010) to produce a lengthening effect in time bisection. This aversive stimulus was an acoustic signal that produces a startle reflex characteristic of a primitive defensive reaction (Hillman et al., 2005). It also increases the level of arousal and fear, as has been demonstrated by Droit-Volet et al. (2010) using both physiological measures (skin conductance responses, SCR) and self-assessment reports (i.e., self-assessment Manikin, SAM; used by Lang, 1980; see Method). In our study, the participants therefore performed a number or length bisection tasks in which they had to judge whether probe stimuli were more similar to a few/short or a many/long anchor stimulus. These stimuli were presented either non-sequentially or sequentially. In addition, in each bisection task, the participants were given trials in which the probe stimulus was followed by either an aversive or a non-aversive stimulus as well as control trials without any emotional stimulus. Our assumption is that a “lengthening” effect should be observed in number and length bisection for the trials with an aversive stimulus compared to those with no signal or with a non-aversive signal, but only when number and length are presented sequentially.

## METHODS

### PARTICIPANTS

Eighty undergraduate psychology students (mean age = 19.34, *SD* = 1.29, 71 females) from Blaise Pascal University in Clermont-Ferrand, France, participated in this experiment in return for course credits. All students gave written informed consent to participate to this experiment following the ethical principles of the declaration of Helsinki.

### APPARATUS

The participants sat wearing headphones in a quiet room in front of a PC. The e-prime program (1.2. Psychology Software Tools, Pittsburg, PA, USA) controlled the experimental events and recorded the data. The material was similar to that used by Droit-Volet (2010) and Droit-Volet et al. (2003). In the non-sequential condition, the stimulus to be judged was a set of black dots for number bisection, and a black line for length bisection. As there were 7 probe durations, the probe stimuli consisted of 8, 10, 12, 14, 16, 18, and 20 dots for number and a line of 8, 10, 12, 14, 16 18, and 20 cm for length. The presentation duration of these stimuli was randomly selected between 1.2 and 4.0 s. In the number task, the spatial arrangement of the dots on the computer screen was also randomly determined. In the sequential condition, the stimulus was a series of successive sets of dots or small lines (number vs. length bisection) which the participants had to accumulate together in order to judge the total number of dots (8, 10, 12, 14, 16, 18, and 20) or the total length (8, 10, 12, 14, 16 18, and 20 cm; for a schematic diagram of the experimental procedure, see Droit-Volet (2010), p. 128). For sequential number bisection, the number of sets of dots was randomly chosen between the values 2 and 5, with the number of dots per set varying randomly among a panel of values depending on the probe stimulus value. For instance, a sequence representing the number 8 could consist of a series of 5 successive sets with 2, 2, 1, 1, and 2 dots, respectively, or a series of 3 sets with 2, 2, and 4 dots, respectively. The number 20 could consist of a series of 5 sets with 4, 3, 2, 5, and 6 dots, or a series of 3 sets with 6, 7, 7 dots or 10, 2, 8 dots. Similarly, for sequential length bisection, the number of lines was randomly chosen between the values 2 and 5, with the length of lines randomly varying among a panel of values depending on the probe stimulus value. For instance, a sequence representing the length 8 cm could consist of a series of 5 successive lines of 3, 1, 1, 1, and 2 cm, respectively, or a series of 3 lines of 2, 1, and 5 cm. The length 20 cm could consist of 5 lines of 3, 5, 2, 4, 6 cm, respectively, or of 2 lines of 8 and 12 cm. The temporal interval between two sets of dots or two lines as well as the presentation duration of each set of dots or each line were randomly selected between the values 0.4 and 1.10 s, with a total sequence duration from 1.2 to 4.0 s. All the stimuli were presented in the center of the computer screen. The participants gave their responses (few/short vs. many/long) by pressing one of two keys (“d” and “k”) on the computer keyboard.

The emotion-inducing stimuli (aversive vs. non-aversive) were acoustic signals delivered binaurally via calibrated headphones. The aversive stimulus was a 50-ms burst of 95 dB white noise with an instantaneous rise time that produced a startle reflex characteristic of a primitive defensive reaction (Hillman et al., 2005). The non-aversive stimulus was a simple beep of 50 dB lasting for 50 ms. These stimuli had previously been tested by Droit-Volet et al. (2010) using both physiological indexes (SCR) and self-assessment reports. It has been demonstrated that the expectation of this aversive stimulus, which produces a mild pain in the ears, is associated with a significant increase in SCR amplitude compared to the expectation of the non-aversive stimulus. In addition, the aversive stimulus is rated on the SAM scale (Lang, 1980) as being of negative valence and highly arousing, with a mean of 7.25 on a 9-point scale, compared to the non-aversive stimulus (mean arousal = 3.5). On emotional scales ranging from 1 (“I don’t feel”) to 6 (“I strongly feel”), this aversive signal has also been judged to produce more fear (3.25) and anger (3.45) than the non-aversive stimulus (0.05 and 0.35, respectively).

### PROCEDURE

The participants were assigned to 4 groups as a function of the bisection task (number vs. length) and the modality of stimulus presentation (sequential vs.** non-sequential). Except for the nature of stimuli to be judged, the task was the same in the different groups. In each bisection task, the participants were initially presented with the two anchor stimuli, i.e., the few/short and the many/long anchor stimuli, five times each. The few/short anchor stimulus had the value 8 (8 n and 8 cm) and the many/long anchor stimulus had the value 20 (20 n and 20 cm). The participants were then presented with 7 probe stimuli (8, 10, 12, 14, 16, 18, 20) and their task was to judge whether the probe stimulus was more similar to the few/short anchor stimulus or to the many/long anchor stimulus by pressing the corresponding key on the keyboard. The key-press order was counterbalanced. In addition, before each trial, a sign indicated whether the stimulus was to be followed by a pleasant or unpleasant sound or by silence. The aversive or non-aversive acoustic signal was delivered just 50 ms after the end of the probe stimulus. The participants completed 8 blocks of 21 trials (168 trials), i.e., one for each of the seven probe stimuli presented in each of the three conditions (aversive, non-aversive, and control). The trials were presented randomly within each block, with an inter-trial interval of either 1.5 or 2.0 s. Each trial started when the participants pressed the spacebar after the word “ready.” The sign then appeared for 50 ms in the center of the computer screen, followed 200 ms later by the probe stimulus (see Apparatus). Then, after a 100-ms interval, the word “response” was presented and the participants responded. Depending on the type of trial, a 50-ms acoustic signal either was or was not delivered 50 ms after the probe stimulus during this 100-ms interval. In addition, the participants were instructed not to count because this distorts the scientific data and were told to continuously repeat aloud “Bla” to prevent verbal counting (Gallistel and Gelman, 2000; Vandierendonck et al., 2004; Rattat and Droit-Volet, 2012).

## RESULTS

**FIGURE 1:**
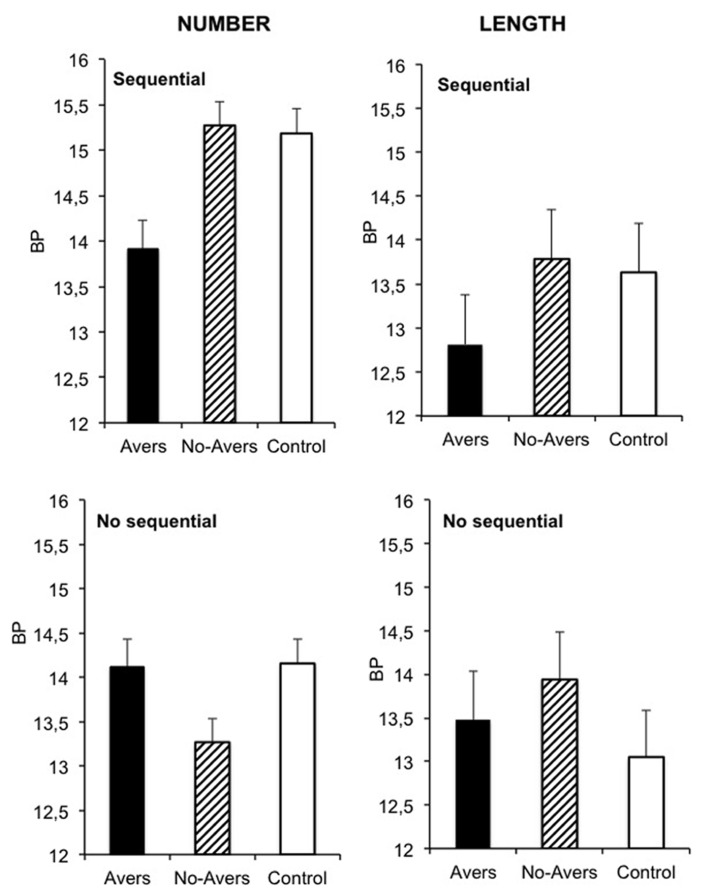
**Mean bisection point for the number and length bisection in the aversive, the non-aversive and the control condition for the sequential and the non-sequential stimulus presentation**.

**Figure 1** shows the bisection point (BP), also called the point of subjective equality. This is the stimulus for which the participants respond “few/short” as often as “many/long” [*p*(many/long) = 0.50]. A low BP value indicates that the participants more frequently respond many/long for the same stimulus, a finding that is consistent with a “lengthening” effect. For each participant, a BP value was calculated from slope and intercept parameters that were obtained by fitting the logistic function from the SPSS program (SPSS version 6 for Windows and Macintosh) to his/her individual psychometric function. The logistic fit of the individual function was not significant for 3 participants in length bisection (one in the non-sequential and two in the sequential presentation condition). These subjects were therefore excluded from subsequent analyses. An overall ANOVA was initially performed on the BP, with the type of quantity (number vs. length) and the sequentiality of the presentation (sequential vs. non-sequential) as between-subjects factors, and emotion (aversive, non-aversive, and control) as within-subjects factor. The ANOVA showed a significant main effect of emotion, *F*(2,146) = 5.36, *p* = 0.006, and of type of quantity, *F*(1,73) = 4.91, *p* = 0.03, while the main effect of sequentiality of presentation was not significant, *F*(1,73) = 1.20, *p* = 0.28. There was also no significant interaction between type of quantity and sequentiality of presentation, *F*(1,73) = 1.71, *p* = 0.20. However, there was a 3-way interaction between emotion, type of quantity and sequentiality of presentation, *F*(2,146) = 4.66, *p* = 0.01, with a significant emotion x quantity type, *F*(2,146) = 4.11, *p* = 0.02, and emotion × sequentiality of presentation interaction, *F*(2,146) = 11.07, *p* = 0.0001. We therefore analyzed the effect of emotion in each condition taken separately.

In the case of number bisection, the effect of emotion was significant in the sequential presentation condition, *F*(2,38) = 24.50, *p* = 0.0001. The BP was significantly lower in the aversive (M = 13.92, *SE* = 0.31) than in the non-aversive (M = 15.27, *SE* = 0.27), *t*(19) = 5.34, *p* = 0.0001, or the control condition (M = 15.19, *SE* = 0.27), *t*(19) = 4.80, *p* = 0.0001, while the non-aversive and control conditions resulted in similar BP values, *t*(19) = 1.07, *p* = 0.30. This indicates that when the numerical quantity was presented sequentially, number was judged greater in the aversive condition than in the other conditions. Emotion also reached significance in the non-sequential presentation condition of the number bisection task, *F*(2,38) = 5.92, *p* = 0.006. However, contrary to the sequential condition, the BP was not lower but higher in the aversive condition (M = 14.12, *SE* = 0.31) than in the non-aversive condition (M = 13.27, *SE* = 0.27), *t*(19) = 4.91, *p* = 0.0001, suggesting that the quantity was judged relatively smaller. However, the aversive BP value did not significantly differ from the control BP (M = 14.16, *SE* = 0.28), *t*(19) = 0.11, *p* = 0.91.

In the case of length bisection, the effect of emotion was significant for sequential presentation, *F*(1,34) = 3.057, *p* = 0.04, while it failed to reach significance for non-sequential presentation, *F*(2,70) = 2.85, *p* = 0.08. Indeed, no significant difference was obtained between the aversive and the other conditions when the length was not presented sequentially (all *p* > 0.05). In other words, it was only in the sequential presentation condition that emotion distorted the judgment of length. The BP was indeed significantly lower in the aversive (M = 12.81, *SE* = 0.57) than in the control condition (M = 13.63, *SE* = 0.56), *t*(17) = 2.19, *p* = 0.04, and was very nearly significantly lower than in the non-aversive condition (M = 13.79, *SE* = 0.56), *t*(17) = 1.96, *p* = 0.067. No difference was observed between the non-aversive BP and the control BP, *t*(17) = 0.56, *p* = 0.58.

In addition, we performed the same analyses on the Weber ratio (WR), which constitutes an index of sensitivity (**Table 1**), i.e., |S(*p*(many/long = 0.75 - |S(*p*(many/long) = 0.25|/ 2. However, the overall ANOVA on the WR with the same factors as those previously reported did not show any significant effect (all *p* > 0.10). This finding is consistent with the results of most studies of time perception by suggesting that emotion produces distortions in the evaluation of quantities, but without affecting the sensitivity.

**Table 1:** Mean and standard error of Weber ratios for the number and the length bisection in the sequential and the non-sequential presentation for the aversive, non-aversive and control trials.

		Aversive		Non-aversive		Control	
		M	*SE*	M	*SE*	M	*SE*
Number	Sequential	0.18	*0.01*	0.22	*0.06*	0.12	*0.04*
	No sequential	0.10	*0.01*	0.18	*0.06*	0.19	*0.04*
Length	Sequential	0.17	*0.02*	0.14	*0.02*	0.16	*0.03*
	No sequential	0.12	*0.02*	0.12	*0.02*	0.14	*0.03*

## DISCUSSION

The present study on number and length bisection used the same emotional context (threatening signal) as that used by Droit-Volet et al. (2010) in temporal bisection. The results showed that, in the same way as for the judgment of time, the judgment of number and length was distorted, with the BP value being lower in the aversive than in the non-aversive or the control condition. The number was thus judged greater and the length longer in a threatening context. However, our results also revealed that this distortion in the judgment of non-temporal quantities only occurred in response to the sequential presentation of quantities. Indeed, when the participants simply had to judge a set of dots or the length of a line (non-sequential stimulus presentation), the expectation of the threatening stimulus did not produce a “lengthening” effect. In this condition, no effect was observed in the length or number bisection task, except for a shortening effect on the trials with a non-aversive stimulus compared to those with an aversive or with no stimulus. By presenting emotional faces before the to-be-estimated stimulus, Young and Cordes (2012) not only found this opposite pattern in numerical bisection but also replicated the lengthening effect in time bisection. These authors explained their results in terms of specific effects of emotion on visual attention in the case of number judgments. They thus argued that different mechanisms underpin the processing of number and time. However, as our study suggests, when the presentation of quantities shared a common property with time, i.e., a dynamic flow of information that the participants had to capture and keep track of in memory, a similar lengthening effect of emotion on judgments occurred and did so irrespective of the magnitude to be judged.

Further research is required to understand the mechanisms underlying this distortion of sequentially presented magnitudes. Nevertheless, our results demonstrate that the lengthening effect observed in an emotional context (highly arousing) is not specific to time judgment, and, consequently, to a mechanism dedicated to the processing of time. The comparison in our study between the sequential and the non-sequential presentation of quantities also suggests that the emotion-related distortions in the magnitude judgments were not specific to the processing of number and length *per se*. When number and length were not presented sequentially, no emotional effect emerged. This is consistent with the results of Droit-Volet’s (2010) study showing that a fast click train produced a subjective lengthening effect only when the quantities were presented sequentially. The emotional distortion in magnitude judgment therefore appears to be due to modifications to the dynamic processing of sequentially presented information or in other words, the processing of the incoming information flow that is a common characteristic of time and of numerosity and length when these are presented sequentially.

As early as 1983, Meck and Church (1983) suggested that the processing of time and number is based on a common pacemaker-accumulator mechanism. According to these authors, the main difference between counting and timing resides in the functioning of the attention-controlled switch connecting the pacemaker to the accumulator which would operate in an “event mode” for number (a brief closing–opening of the switch each time an unit is counted) and in a “run mode” for time (opening and closing of the switch at the onset and offset of the stimulus, respectively). Several studies have shown that emotion influences the encoding of threatening events by facilitating their perception through attentional processes (Phelps, 2006; Phelps et al., 2006; Phelps and Sharot, 2008). For example, emotion enhances the detection of emotion-eliciting items in situations where attentional resources are limited. Other studies have shown that threatening cues (i.e., faces expressing fear) facilitate not only the detection of emotional items, but also that of neutral items presented in the same visual field, i.e., in “close spatial and temporal proximity” (for a review, see Pourtois and Vuilleumier, 2006). According to Pourtois and Vuilleumier (2006), emotions thus facilitate the orientation of attention (both spatial and temporal) to individual items in a sequence of items. In this theoretical perspective, it seems plausible that emotion might have improved the accumulation of the incoming information provided by a sequence of numbers or lengths. Using a variety of temporal tasks, Gil and Droit-Volet (2011) have demonstrated that the emotion-related lengthening effect observed in the judgment of time is indeed related to more accurate timing, with the estimated durations being closer to the target duration. The emotional lengthening effect would thus appear to result from a reduction in the loss of temporal units.

The mechanisms underlying the effect of emotion (negative-highly arousing) on the perception of time are currently a matter of debate (for a recent review, see Droit-Volet, 2013; Droit-Volet et al., 2013). Some researchers have suggested that emotion increases the amount of attention allocated to the processing of time (Meck and Macdonald, 2007; Lui et al., 2011; Smith et al., 2011). The faster detection of emotional stimuli causes the attentional switch to close earlier. When the switch closes early, more units are accumulated and the ensuing period is judged to be longer. In this perspective, and also in line with the event-mode model (Meck and Church, 1983), the lengthening effect obtained with the sequential presentation of non-temporal quantities would be due to the fact that the switch closes more frequently (event mode) if an aversive stimulus is expected, thus reducing the loss of incoming information in the number or length sequence. Other researchers, however, have suggested that emotions increase the arousal level which, in turn, speeds up the internal clock during the overall processing of time (e.g., Droit-Volet et al., 2004; Droit-Volet and Meck, 2007; Bar-Haim et al., 2010; Mella et al., 2010; Tipples, 2011; Gil and Droit-Volet, 2012). When the clock runs faster, more units are accumulated and time is also judged longer. However, our findings with non-temporal quantities revealed that the lengthening effect is not specific to an internal clock system. Consequently, the increase in arousal with emotion appears to speed up a general information processing system rather than a specific internal clock system. In the case of dynamic sequences of information, this system might take the form of an information accumulation system in working memory. Several studies of working memory have shown that the increase in information processing speed reduces the risk of loss of information in working memory (e.g., Kail and Salthouse, 1994; Baddeley, 2012). However, the nature of accumulated units is probably different for temporal information and non-temporal quantities as those used in our study. As suggested a reviewer, our findings on non-temporal quantities do not allow us to reject the hypothesis that high-arousing emotion could accelerate the speed of a general information accumulation system for non-temporal quantities, while they could accelerate the speed of both this general information accumulation system and a specific internal clock system for the processing of durations.

In addition, the results of our study do not allow us to decide between these two processes, namely attention and arousal-related processing speed. It is indeed particularly difficult to distinguish between an arousal-related effect linked to an increase in information accumulation speed, and an attention-related effect linked to an increase in the attentional capture of incoming information. As explained by Paus (2000), these two processes are closely interrelated. Indeed, in threatening situations, the organism mobilizes attentional resources to detect forthcoming stimuli (vigilant attention system; e.g., Vuilleumier et al., 2003; Ledoux, 2012). And this state of alarm also triggers an array of changes in the autonomic (cardiovascular, respiratory) and somatic (facial and bodily motor expression) nervous systems that help prepare the organism to act as quickly as possible, i.e., to fight or to flee. Finally both arousal and attention help increase the speed and efficiency of information processing in order to detect potential dangers in the environment and initiate action as quickly as possible. In conclusion, our study suggests that negative emotions such as fear cause the dynamic flow of quantitative information (time, sequence of number or length) to be judged longer and that this is probably due to the improved accumulation of information in working memory. However, the effects of emotion on efficiency of information accumulation system in the judgment of quantities must be further examined. The results of our study mainly show that the lengthening effect induced by emotion is not specific to time.

## Conflict of Interest Statement

The author declares that the research was conducted in the absence of any commercial or financial relationships that could be construed as a potential conflict of interest.
